# Primary cilia and their role in preeclampsia

**DOI:** 10.7150/ijms.114433

**Published:** 2025-07-10

**Authors:** Lingyun Liao, Rong Zhou, Min Liu

**Keywords:** preeclampsia, primary cilia, placenta, signaling transduction, vascular development.

## Abstract

Preeclampsia is a hypertensive disorder of pregnancy characterized by chronic placental ischemia and systemic maternal organ damage. The placenta is rich in blood vessels containing various types of cells, and preeclampsia is now widely accepted as a placenta-derived disease. Although the primary cilium regulates many diseases, its role in preeclampsia has not been comprehensively studied. Therefore, we conducted a review to provide valuable insights into the current understanding of the pathogenesis of preeclampsia, especially as related to the primary cilium, and to provide direction for new research objectives. Primary cilia are sensory microtubule-based organelles that translate extracellular clues into intracellular signals for molecular and cellular responses. They are crucial for the regulation of vascular development and have various mechanisms, such as epithelial-to-mesenchymal transition, reaction to mechanical stress, and mediation of signal transduction. Dysfunction of the primary cilia contributes to aberrant fluid sensing or signal transduction, resulting in vascular disorders. Here, we summarize that angiogenic factors or inflammatory cytokines change the biological behaviors and functions of placental cells in preeclampsia by altering the length or signaling function of primary cilia. We further outline the role of the primary cilia in vascular endothelial function and other female reproductive disorders. Further research is needed to understand the underlying mechanisms and clinical treatment of primary ciliary deficiency in preeclampsia.

## Introduction

Preeclampsia is defined as new-onset hypertension arising after 20 weeks of gestation, often accompanied by proteinuria. Preeclampsia is a pregnancy disorder that damages multiple systems and presents as a well-accepted clinical syndrome characterized by major cardiovascular manifestations attributable to endothelial dysfunction, systemic inflammation, and generalized vasoconstriction, resulting in hypertension and multi-organ hypoperfusion 1. Preeclampsia is responsible for the mortality of approximately 76,000 women and 500,000 babies worldwide each year 2. In some economically backward areas, such as Africa, preeclampsia occurs in 10% of pregnancies, which is significantly higher than the global average of approximately 2% 2. Clinically, expectant management is usually accepted to prolong gestation to decrease neonatal mortality; however, delivery is the only effective treatment for preeclampsia 3. Moreover, preeclampsia can have long-lasting consequences for both the mother and fetus, including cardiovascular disease 4, postpartum depression 5, and neurological disability 6.

The primary cilium, a hair-like structure protruding from the cell surface, enables cells to respond to extracellular environmental cues through the corresponding receptors on their surface membrane. It acts as a hub for cell signaling and is present in almost all vertebrate cells 7. Anatomically, the assembly and disassembly of the primary cilia are closely associated with cell-cycle progression; therefore, cilia are important for cell proliferation. Dysregulation of structural and functional cilia causes multiple severe human disorders, collectively termed ciliopathies 8. Although it has been several decades since the primary cilium was found to regulate many diseases (including obesity, nephronophthisis, mental retardation, vascular diseases), its role in preeclampsia has not been studied in depth. Hence, a good understanding of ciliary structure and functional ciliary proteins is vital to investigate how ciliary dysfunction contributes to preeclampsia. This review provides valuable insights into the current understanding of the pathogenesis of preeclampsia and provides direction for new research objectives.

## Structure and function of primary cilia

The primary cilium is a non-motile organelle with an antenna-like structure extending from cell membranes, which mainly includes the basal body and the axon portion. The basal body is derived from the mother centriole of the centrosome, and its conversion indicates that the primary cilium begins to form depending on the completion of cell division. The transition fibers (TF) of the basal body anchor the mother centriole to the ciliary membrane, and the distal end of the basal body regulates vital aspects of ciliary biogenesis and function 8, 9. The proximal end of the cilium above the basal body is called the transition zone, which controls the entrance and exit of ciliary proteins along with the TF and contributes to the compartmentalization of the organelle 10. The axoneme, which plays an important role in sensing the surrounding microenvironment, grows from the basal body, as the core of primary cilium, and is mainly assembled by a ring of “9+0” pairs of microtubule doublets that run longitudinally through the organelle. This cilium is distinguished from the motile “9 + 2” cilia, which have an additional two dynein-associated central microtubules, permitting motion 11.

The TF are essential for the recruitment of intraflagellar transport (IFT) system components, primary cilia ferry receptors, and other proteins into and out of the cilium through IFT particles. Cilia length, in part controlled by the activity of the IFT, is a parameter for adequate function and length of primary cilia. Inactivation of IFT proteins usually results in a deficiency of primary cilia in the affected cell; for example, IFT88 knockout targets in the cilia can cause defective ciliogenesis and tubular mitochondrial damage in proximal tubular cells in acute kidney injury 12. IFT proteins (IFT-A and IFT-B complexes) form multi-subunit complexes in concert with BBSome, motors (kinesin and dynein), and specific ciliary cargo proteins for transport during anterograde or retrograde IFT 13. BBSomes, a complex of BBS proteins, function as IFT adapters for the transport of ciliary proteins. BBSomes also regulate the trafficking of certain membrane proteins into and out of the cilia and the export of signaling proteins 14, 15. Kinesin motors facilitate anterograde IFT from the cytoplasm to the ciliary tip, while cytoplasmic dynein motors mediate retrograde transport from the ciliary tip to the cytoplasm 16. All these IFT build and affect axoneme microtubule assembly by supplying axonemal components to the ciliary tip. The axoneme leads to IFT and intraflagellar signaling transport along the ciliary shaft using the cilioplasm. In addition, the ciliary membrane is attached to the cell membrane but has unique lipid and receptor compositions that allow the cilia to detect changes in the extracellular environment and convey signals to cells to regulate a variety of cellular, developmental, and physiological processes 17, 18. An emerging class of functions involves signaling receptors and/or effectors delivered to the ciliary axoneme by specialized transport machinery and sequestered to regulate morphogenetic signaling pathways.

## Signaling transduction in primary cilia

Most primary cilia serve in signal transduction; in turn, signaling is dependent on a highly specialized primary cilium. Primary cilia play a critical role in sensing extracellular stimuli, and stimulation leads to intracellular signaling transduction 7.

### Hedgehog signaling pathway

The Hedgehog (Hh) signaling pathway plays an important role in cell growth, differentiation, and intercellular communication. The Hh signaling pathway can be divided into two different pathways: classic and atypical. In vertebrates, canonical Hh signaling (Hh-Ptch-Smo-Gli) is triggered by one of three Hh proteins [Sonic hedgehog (SHH), Indian hedgehog, or Desert hedgehog], which act as ligands and ultimately lead to changes in signal transduction by altering the balance of activators and repressors of Gli family zinc finger transcription factors. In the absence of HH ligands, the receptor Patched-1 localizes to the primary cilia and reduces the accumulation and activation of Smoothened (SMO) protein on the ciliary membrane. Upon binding of HH ligands to PTCH-1, Patched-1 is endocytosed, relieving the repression of SMO 19. SMO, a transmembrane protein of the G-protein-coupled receptor (GPCRs) superfamily that acts downstream of Patched-1, moves to the tip of the primary cilia and releases the Gli activator 20. In the non-classical pathway, Hh relies only on a certain point in the signaling pathway to conduct the signal 21. Note that once primary cilia are lost, SMO is unable to activate Gli transcription factors, and primary cilia are required for Gli repressor formation in the absence of the Hh ligand. Thus, the regulatory effects of primary cilia and Hh signaling are reciprocal.

### Wnt signaling pathway

The Wnt signaling pathway is generally divided into canonical (Wnt/β-catenin) and noncanonical (Wnt/planar cell polarity [PCP]) pathways. Wnt cascades are usually initiated by the binding of a secreted Wnt ligand to a Frizzled family receptor, which leads to the activation of its intracellular binding partner Dishevelled 22. Primary cilia negatively regulate canonical Wnt signaling, and the presence of cilia represses the nuclear accumulation of β-catenin, which is a hallmark of the inactivation of canonical Wnt signaling 23. Furthermore, another view is that the primary cilia act as a molecular switch between canonical and non-canonical Wnt signaling activity. The non-canonical PCP pathway is believed to play a role in maintaining the coordinated beating of cilia and normal cellular homeostasis 24, 25. Some key components that regulate the PCP pathway are localized to the cilium. For example, deficiency of Celsr1, a PCP gene, results in ciliary motility that is not orchestrated along the ovarian-uterine axis, and the transport ability of beating cilia is impaired 26.

Strikingly, cilia are not required for Drosophila Hh and Wnt signal transduction 19. However, it is controversial whether cilia are required for Hh and Wnt signaling in non-mammalian vertebrates such as zebrafish. Huang et al. indicated that cilia are not essential for Wnt signaling in zebrafish and found that MZovl mutant cells not only lack the ciliary axoneme but have whole basal bodies, which might mediate Wnt signaling in the absence of cilia 27. Thus, it is conceivable that the ciliary axoneme and basal body are two distinct signaling organelles with discrete functions.

### Other signaling pathways

Primary cilia also participate in receptor tyrosine kinases (RTKs), GPCRs, Notch, transforming growth factor beta (TGF-β), mTOR, and other signaling transduction. RTKs comprise a large family of tyrosine kinases with similar structural organization, including an extracellular ligand-binding domain, which are high-affinity cell surface receptors for many peptide growth factors, cytokines, and hormones. RTKs bind to ligands and undergo phosphorylation at intracellular tyrosine residues, thereby activating downstream signaling pathways, such as extracellular signal-related kinase (ERK), MAPK, PI3K-AKT, and phospholipase Cγ 28. For example, platelet-derived growth factor receptor-alpha (PDGFRα) is up-regulated during ciliogenesis, and ciliary localization of PDGFRα is required for its appropriate ligand-mediated activation by PDGF-AA 29. The primary cilia of endothelial cells regulate islet vascularization and vascular barrier function via the vascular endothelial growth factor (VEGF)-A/VEGFR2 signaling pathway 30.

The Notch signaling pathway requires a signal-sending cell and a receiving cell to participate, and the signal-sending cells harbor ligands for receiving cells containing Notch receptors located on the primary cilium. Notch signaling components generally act downstream of primary cilium 31. Notch signaling was first identified as requiring whole and functional primary cilia to initiate differentiation of embryonic keratinocytes 32. Compared to the wild type, cilia-impaired (knockout of the cilia-building genes FSD1, KIF3A, PKD2, and IFT88) zebrafish embryos have downregulated Notch signaling 33. However, another study provided evidence that loss of BBS expression in zebrafish or cultured cells resulted in reduced localization of the Notch receptor to the cilium but enhanced activation of the Notch pathway, which might be caused by improperly degraded Notch receptors when BBSome proteins are depleted 34.

The primary cilium is needed for activation of the TGF-β pathway, and dysregulation of TGF-β signaling or cilia has been linked to several organ pathologies 35. TGF-β plays its role by binding to the TGF-β receptor (TGF-βR) on the tip of the cilium, which triggers a signaling cascade and the activation or nuclear translocation of SMADs (SMAD2, SMAD3, and SMAD4), thus upregulating the target gene expression 36. IFT80 deletion downregulated the TGF-β signaling pathway by inhibiting the expression of TGF-βI, TGF-βR, and phosphorylation of SMAD2/3, thereby causing the pheno primary cilium type of reduced chondrocyte proliferation, cilia assembly, and chondrogenic differentiation 37. In another study, however, TGF-β stimulation caused shortening and even loss of primary cilia in osteoblasts by inhibiting bone morphogenic proteins 2 and 7 38. Therefore, there is a mutual regulatory effect between the TGF signaling pathway and primary cilia.

## Brief advances of preeclampsia

The pathogenesis of preeclampsia is still difficult to reach a consensus on, but the two-stage model is the most widely accepted 39. During placental implantation in early pregnancy, extravillous trophoblasts (EVTs) are differentiated from cytotrophoblast cells, which adhere to the maternal decidua, invade the decidua, and replace part of the endothelial cells and vascular smooth muscle cells on the wall of the uterine spiral artery, a process known as spiral artery remodeling 40. In this process, the epithelial to mesenchymal transition (EMT) of EVTs can enhance their migration and invasion capacity by losing proliferation, tight epithelial assembly, and apical basal polarity; thus, the EMT of EVTs is vital for maternal decidual invasion 41. The remodeling process results in the loss of endothelial cells, low contraction resistance, and a large diameter uterine artery, which delivers abundant maternal blood flow to the placenta to support fetal development. Conversely, failure of spiral artery remodeling can result in placental ischemia and inadequate placental perfusion 42. Impaired spiral artery remodeling is the first stage in the two-stage model.

Uterine spiral artery remodeling disorder is the pathological basis of preeclampsia and the origin of secondary placental and systemic vascular endothelial dysfunction. Trophoblast cells are in a constant hypoxic microenvironment and undergo oxidative stress when placental perfusion is compromised. Oxidative stress can increase reactive oxygen species production and catabolism of the vasodilator nitric oxide, thus generating arteriolar spasms and further placental ischemia, which exacerbates oxidative stress 43. This chain reaction could increase the anti-angiogenic factors and vascular intimal injury in the intrauterine environment and maternal circulation, inducing an excessive inflammatory response 44. Subsequently, an imbalance in angiogenic and anti-angiogenic factors, such as vascular endothelial growth factor (VEGF) and placental growth factor (PlGF), triggers endothelial dysfunction in preeclampsia 45. Systemic vascular inflammation and endothelial dysfunction constitute the second stage of the two-stage model.

## Primary cilia-dependent vascular endothelial function

Preeclampsia is a disease of placental origin, and the placenta is rich in blood vessels. The vascular endothelium is currently recognized as an essential homeostatic organ that controls vascular tone and structure and is partly dependent on primary cilia. Endothelial primary cilia have been found in mouse arteries, blood vessels from the human placenta *ex vivo*, and brain vasculature 46, 47. An important endothelial function is to sense mechanical stimuli (e.g., shear stress and pressure) and/or chemical stimuli (e.g., vasoactive substances and hormones) 46. The primary cilium functions as a mechanosensor that senses fluid flow in various cell types 48. Its length is positively correlated with mechanosensory action. Blood vessels with relatively low shear stress from physiological blood flow have longer cilia, while blood vessels with a high fluid force are devoid of cilia or have very short cilia owing to the fluid force eliciting cilia bending 49. The lining of the inner surface of vascular blood vessels consists of endothelial cells with primary cilia protrusions, which can sense changes in blood velocity and pressure and convert these mechanical signals into changes in vascular smooth muscle tone 50. Polycystin-1 (PC1) and PC2 reportedly form a mechanosensory complex and are assembled in a stoichiometry of 3 PC2 for every PC1 molecule in the primary cilia; these complex tests the bending of the cilia by the fluid flow 51. The absence or lack of flow, as well as the loss or dysfunction of the cilia, PC1, or PC2, decreases Ca2+ influx and activates intramembrane proteolysis, which allows STAT6 and its coactivator P100, in a complex bound to the PC1 tail, to translocate to the nucleus and stimulate transcription, resulting in uncontrolled cell proliferation and cyst formation 52. Moreover, the PC1 tail triggers several cytokines and growth factor signaling, amplifying the cellular response and potentially leading to an increase in cell proliferation, activation of endothelial nitric oxide synthase (eNOS) and subsequent vasodilation 46, 53.

Various vascular diseases can be caused by the length of vascular endothelial cilia and abnormal signal transduction functions. Gupta et al. suggested that cilia and ciliary proteins in circulation under various altered-flow conditions could serve as biomarkers of damaged endothelia 54. The endothelial RGS12 protein can promote ciliogenesis and cilia prolongation, thereby enhancing angiogenesis. RGS12 may be a potential anti-angiogenic drug target for inflammatory arthritis 55. The loss of endothelial cilia increases inflammatory gene expression and downregulates eNOS activity, revealing that endothelial cilia restrain proatherosclerotic signaling 56. Considering that endothelial dysfunction in preeclampsia predominates in clinical syndromes and the primary cilia in vascular development, we speculate that ciliary shortages or altered ciliary proteins also mediate these changes in blood vessels in preeclamptic placentae.

## Primary cilia in preeclampsia

A growing body of evidence suggests that cilia are crucial for human embryonic development and the maintenance of pregnancy. After embryo implantation, cilia first appear on E5.5, and the ciliated cell numbers increase rapidly during the second trimester and throughout the embryonic stage 57. Primary cilia were only detected in a few EVTs in the proximal cell columns of the placental tissue during the first and second trimesters. This may be related to the fact that the trophoblast cell column has a high density of cellular composition. Alternatively, only a small fraction of trophoblasts is ciliated for a specific period and then disappear after completing their specialized functions 58. Term placentae show barely ciliated trophoblasts, which are likely associated with a different hormonal milieu in the last trimester 59.

**Figure 1:**
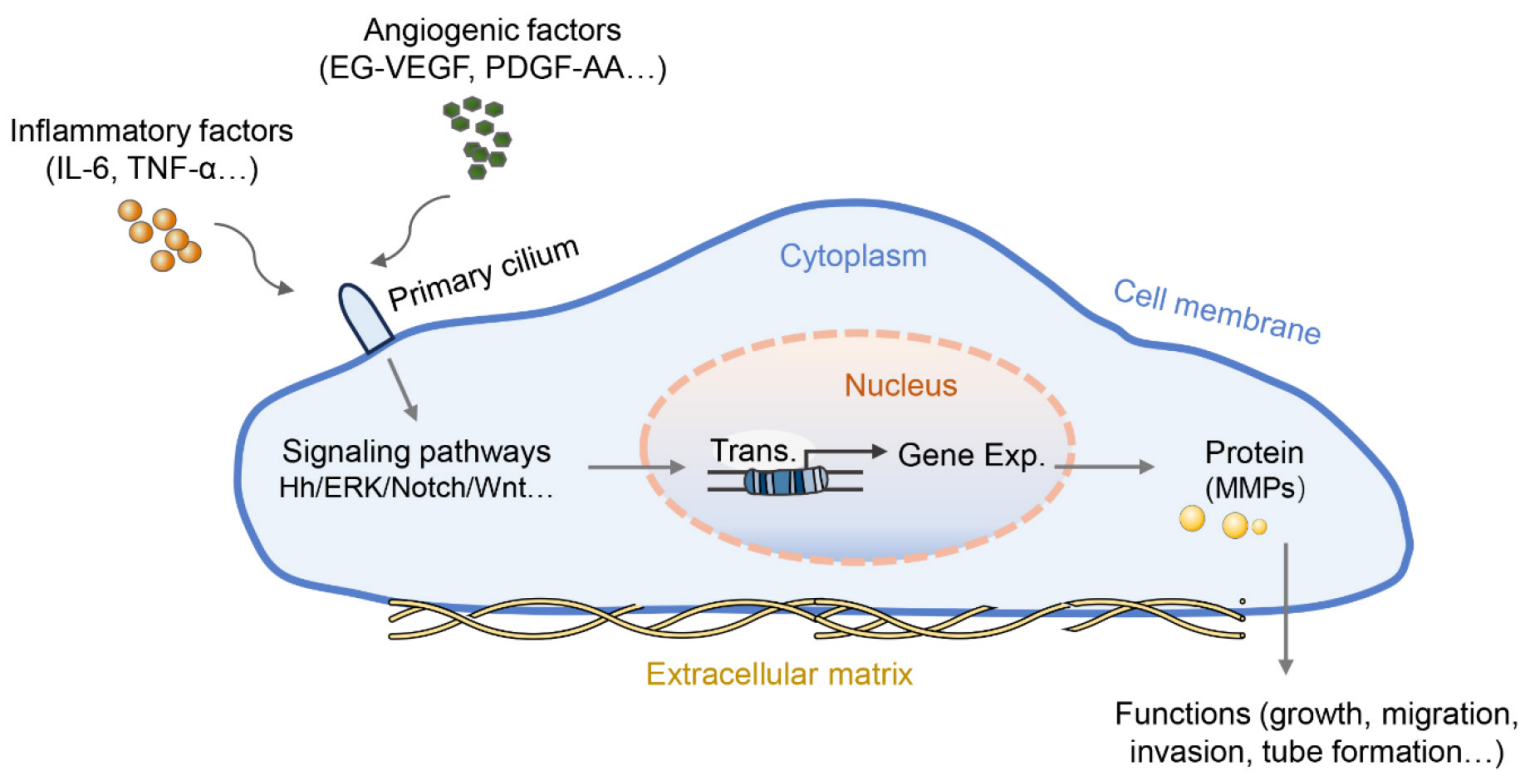
The role of primary cilia in preeclamptic placental cells. Some angiogenic factors or inflammatory cytokines can bind to their corresponding receptors located on the primary cilia, blocking the signaling transduction served by primary cilia. The blocked signaling pathway further abnormally regulates the transcription and protein translation of genes for cell function regulation, such as cell proliferation, migration, tube formation, etc. Abnormalities in primary cilia have been proposed to promote the pathology of preeclampsia.

Wang et al. first reported the role of primary cilia in the placenta with a focus on trophoblast invasion. PROKR1, similar to other GPCRs, is located on the primary cilia in both trophoblast cell lines (HTR-8/SVneo and 3A-subE) and human placental tissue and is required for EG-VEGF-regulated ERK1/2 activation both in the cytoplasm and the base of the primary cilium, MMP expression, and trophoblast cell invasion 60. The follow-up work in this study found that the number of cilia in the placenta of preeclamptic women was significantly lower than that in the normal placenta. In the peripheral blood and placenta of preeclamptic patients, plasma miR-141 and miR-200a suppress the expression of EG-VEGF, downstream ERK/MMP9 signaling, and primary cilia formation, leading to insufficient trophoblast invasion 61. Similarly, another study reported that PDGF-AA promotes placental choriocarcinoma JAR cell growth; in turn, JAR cells can grow primary cilia where the PDGF-AA receptor is enriched 62. Interestingly, the Hh pathway can induce EMT in human trophoblasts and perform critical functions exclusively mediated by the primary cilium 63. Moreover, treatment with crucial preeclampsia-related inflammatory cytokines (IL-6 and TNF-α) induced defective primary cilia and impaired Hh signaling in trophoblastic cells, thus leading to slowly migrating trophoblasts and intricate tube formation with reduced expression of MMP mRNA 58. Additionally, the shortened cilium length revealed an impairment of the Hh pathway and poor differentiation capacity in chorionic villous stromal cells from term preeclampsia placentas, possibly contributing to the reduced mobility of trophoblastic cells as well as impaired tissue repair or homeostasis in preeclampsia 64. These results suggest that angiogenic factors or inflammatory cytokines regulate the growth, migration, invasion, and tube formation of trophoblasts by altering the length or signaling function of the primary cilia (Figure 1). However, the exact mechanisms by which primary cilia affect the behavior and function of trophoblasts are not fully understood.

## Primary cilia in other female reproductive disorders

Endometrial stromal differentiation and trophoblast invasion are partly determined by the normal morphology and function of cilia, and their abnormalities are two major pathogenic mechanisms of some female reproductive disorders. Pearson-Farr et al. found that subfertile women have proportionately lower ultrastructurally normal cilia, higher frequency of absent dynamin arms or inner arm defects, and lower cilia beat frequency 65. The length of primary cilia and the percentage of ciliated cells was significantly decreased in the decidua, and the percentage of ciliated deciduastromal cells decreased significantly in decidua of patients suffering recurrent abortion 66. Studies have found that mutation in ciliary genes might be associated with pregnancy loss, primary cilia may be involved in spontaneous abortion by affecting uterine decidualization. The heterozygous deleterious mutations affecting DYNC2H1 have been detected in women with recurrent pregnancy loss by whole exomesequencing technology 67, and DYNC2H1 was associated with prenatal or neonatal death in human by regulating ciliogenesis 68. This suggests that gene mutations related to primary ciliary development may be an important cause of miscarriage. This was confirmed in animal experiments that decidualization required primary cilia that increased in the mouse uterine stroma prior to implantation, and impaired embryo implantation and decidualization are the major causes of pregnancy failures 69, 70. Moreover, miR-20b-5p suppressed primary cilia formation and trophoblast invasion by reducing the expression of ATG16L1 and ATG7, and the defective phenotypes could be rescued by aspirin in recurrent abortion 71. The above indicates that the morphological and functional abnormalities of primary cilia are directly related to pregnancy loss.

Improper decidualization has been tightly associated with recurrent implantation failure (RIF). Studies have found that primary cilia are abnormal in the endometrium of RIF patients, aberrant primary cilium induces failed human decidualization through abnormal regulation of PTEN-PI3K-AKT-FOXO1 signaling 72. Hh family members also play vital roles in decidualization and highlight the importance of primary cilia in the endometrial remodeling that is required for pregnancy and that is dysregulated in diseases such as endometriosis 73. The primary cilia not only participate in the aforementioned diseases mediated by decidualization failure, but also regulate other reproductive disorders. Primary cilia regulate the expression of steroidogenic enzymes, thereby promoting progesterone secretion by granulosa cells in mice and ensuring proper luteinization 74. Conversely, progesterone can also significantly increase the rate of ciliated cells and cilia length in stromal cells of endometrial samples and cultured stromal cells 75. Furthermore, IFT88 as a gene required for cilia formation andmaintenance, the synthesis and secretion of estrogen are impaired due to the absence of IFT88 in granulosa cells 76. Therefore, primary cilia may be involved in the regulation of the occurrence of ovari-related diseases.

## Conclusion and future perspectives

The structure and sensory functions of the primary cilia are essential for normal tissue homeostasis and function. Over the past few years, researchers have made tremendous advances in our understanding of the basic cellular and molecular functions of primary cilia. However, the role of the primary cilia in preeclampsia remains unclear. This review summarizes the fact that some types of placental cells with defective cilia may be associated with compromised angiogenesis and failed tissue homeostasis in preeclampsia. Most studies have focused on the influence of certain cytokines on ciliary length or absence in preeclampsia; however, specific molecular mechanisms, such as the formation of primary cilia, fluid flow, and signal transduction, are lacking. Further studies are required to identify additional factors in the blood of preeclamptic patients involved in impaired cilia and to explore the impact of the cilia on other placental cell types, such as endothelial cells, vascular smooth muscle cells, and mesenchymal stem cells. In addition, vascular development is the focus of placental development and the pathology of preeclampsia. Therefore, we have expanded the connection between primary cilia and vascular development. A recent study has indicated that acquired ciliopathies, including preeclampsia, may also respond to dietary lipid therapy 77; however, there is no direct experimental evidence for this. Unfortunately, no studies have identified drugs that can reverse the deficiency of primary cilia in placental cells; thus, more research is warranted to gain basic scientific knowledge into clinical considerations and perspectives.
